# Calcineurin B-Like Proteins CBL4 and CBL10 Mediate Two Independent Salt Tolerance Pathways in *Arabidopsis*

**DOI:** 10.3390/ijms20102421

**Published:** 2019-05-16

**Authors:** Yang Yang, Chi Zhang, Ren-Jie Tang, Hai-Xia Xu, Wen-Zhi Lan, Fugeng Zhao, Sheng Luan

**Affiliations:** 1 Nanjing University-Nanjing Forestry University Joint Institute for Plant Molecular Biology, College of Life Sciences, Nanjing University, Nanjing 210093, China; yangyang_1604@163.com (Y.Y.); zhangchi0919@126.com (C.Z.); lanw@nju.edu.cn (W.-Z.L.); 2 Department of Plant and Microbial Biology, University of California, Berkeley, CA 94720, USA; rjtang@berkeley.edu; 3 College of Agronomy, Henan Agricultural University, Collaborative Innovation Center of Henan Grain Crops, Zhengzhou 450002, China; hauxhx@163.com

**Keywords:** calcium sensor, CBL10, ion homeostasis, salt stress, SOS pathway

## Abstract

In *Arabidopsis*, the salt overly sensitive (SOS) pathway, consisting of calcineurin B-like protein 4 (CBL4/SOS3), CBL-interacting protein kinase 24 (CIPK24/SOS2) and SOS1, has been well defined as a crucial mechanism to control cellular ion homoeostasis by extruding Na^+^ to the extracellular space, thus conferring salt tolerance in plants. CBL10 also plays a critical role in salt tolerance possibly by the activation of Na^+^ compartmentation into the vacuole. However, the functional relationship of the SOS and CBL10-regulated processes remains unclear. Here, we analyzed the genetic interaction between CBL4 and CBL10 and found that the *cbl4 cbl10* double mutant was dramatically more sensitive to salt as compared to the *cbl4* and *cbl10* single mutants, suggesting that CBL4 and CBL10 each directs a different salt-tolerance pathway. Furthermore, the *cbl4 cbl10* and *cipk24 cbl10* double mutants were more sensitive than the *cipk24* single mutant, suggesting that CBL10 directs a process involving CIPK24 and other partners different from the SOS pathway. Although the *cbl4 cbl10, cipk24 cbl10,* and *sos1 cbl10* double mutants showed comparable salt-sensitive phenotype to *sos1* at the whole plant level, they all accumulated much lower Na^+^ as compared to *sos1* under high salt conditions, suggesting that CBL10 regulates additional unknown transport processes that play distinct roles from the SOS1 in Na^+^ homeostasis.

## 1. Introduction

Soil salinity imposes ion toxicity, hyperosmotic stress, and secondary stresses such as oxidative damage and nutritional disorders on plants [1]. Plants have evolved several mechanisms to respond to the harsh environment and adjust their growth under high salt conditions [2]. One critical mechanism involves calcium elevation and calcium-dependent signaling pathways in plant cells [3]. The SOS (salt overly sensitive) pathway represents a calcium-dependent signaling pathway responsible for Na^+^ homeostasis and salt tolerance in *Arabidopsis* [4]. The pathway starts from CBL4 (calcineurin B-like 4), a calcium sensor protein that is supposed to respond to the specific Ca^2+^ signals triggered by excess Na^+^ [5]. The CBL4 protein interacts with a serine/threonine protein kinase (CIPK24) that activates the SOS1, a Na^+^/H^+^ antiporter, leading to the Na^+^ efflux from the cytosol [6,7,8]. In addition to interacting with SOS1 at the plasma membrane, CIPK24 is also reported to regulate the activity of several tonoplast localized transporters by interacting with them, such as the Ca^2+^/H^+^ antiporter [9], the vacuolar V-ATPase [10] and the Na^+^/H^+^ exchanger [11,12]. Additionally, CIPK24 may link flowering time and salt stress response as its activity was regulated by a photoperiodicity and circadian clock switch GIGANTEA (GI) [13]. CBL4 also plays a critical role in the development of lateral roots through the modulation of auxin gradients and maxima in roots under mild salt conditions [14,15].

While CBL4 mainly functions in root tissues, CBL10 appears to be preferentially expressed in the shoots and plays a key role in salt stress tolerance as well [16,17]. Similar to CBL4, CBL10 also physically interacted with CIPK24. However, in contrast to the CBL4-CIPK24 complex at the plasma membrane, the CBL10-CIPK24 interaction was primarily associated with vacuolar compartments [16,18]. As CBL4 and CBL10 cannot replace each other’s functions [17], they must fulfill distinct regulatory functions in the salt stress response of Arabidopsis plants. Furthermore, a recent study has demonstrated that CBL10 is critical for reproductive development under salinity conditions and it functions independently from the SOS pathway [19]. Studies so far have shown that the salt-tolerance function conferred by CBL10 is conserved in *Arabidopsis* [16,17], poplar [20] and tomato [21]. In poplar, two CBL10 homologs have been identified and they serve similar functions [20]. In tomato, a CBL10 homologue has been shown to function in Na^+^/Ca^2+^ homeostasis under salt stress and reactive oxygen species (ROS) signaling in plant immunity [21,22].

The CBL-CIPK pathway is widely accepted as a major mechanism underlying plant response and adaptation to different external stresses that trigger Ca^2+^ signaling events [23,24]. Although several other CBL-CIPK pairs have been implicated in salt tolerance [25,26,27,28], the CBL4- and CBL10-dependent pathways play dominant roles in salt tolerance. In this study, we examined the genetic interaction of the two major salt response pathways directed by CBL4 and CBL10 that mediate salt tolerance by regulating processes at the plasma membrane and intracellular membranes, respectively. Detailed characterization of the *cbl4 cbl10*, *cipk24 cbl10* and *sos1 cbl10* double mutants under salt stress demonstrated that CBL4 and CBL10 fulfill distinct mechanisms of salt tolerance in plants.

## 2. Results

### 2.1. Functional Synergy of CBL4 and CBL10 in Arabidopsis

**Figure 1:**
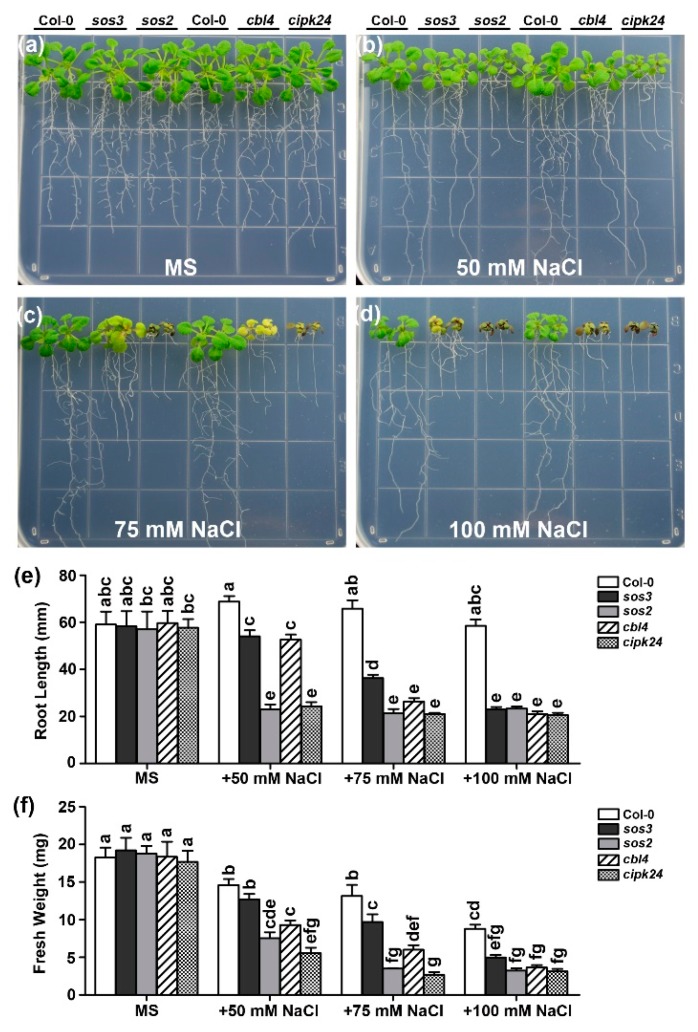
The *cbl4* mutant plants are more sensitive to external Na^+^ than *sos3* mutant plants. (**a–d**) Growth phenotype of Col-0, *sos3*, *sos2*, *cbl4* and *cipk24* under different concentrations of external NaCl. Five-day-old wild type and mutant seedlings were transferred onto the Murashige and Skoog (MS) medium or MS supplemented with 50 mM, 75 mM and 100 mM NaCl. Photographs were taken on the 14th day after the transfer; (**e**) length of primary roots and (**f**) fresh weight of seedlings on the 14th day after the transfer. Data are presented as the mean ± Standard Error(SE) of triplicate experiments. Values labeled with different letters are significantly different (*p* < 0.05).

**Figure 2:**
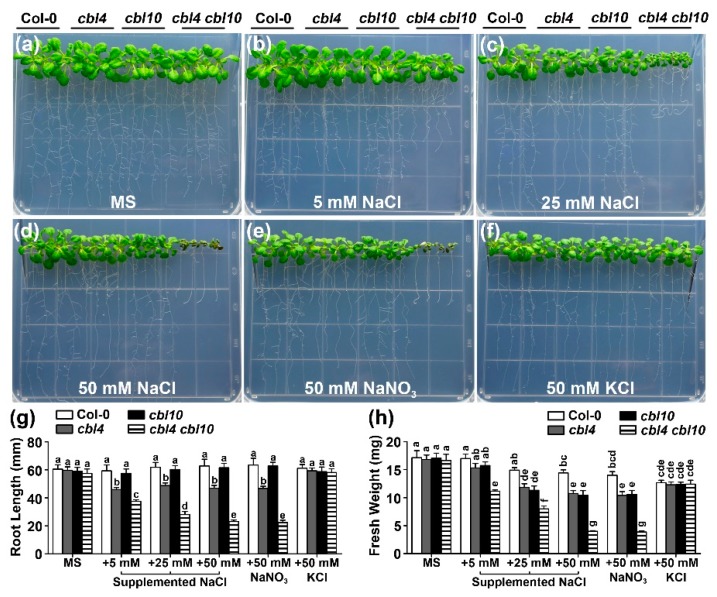
The *cbl4 cbl10* double mutant plants are more sensitive to external Na^+^ than *cbl4* and *cbl10* single mutants. (**a–f**) Growth phenotype of Col-0, *cbl4*, *cbl10* and *cbl4 cbl10* under different concentrations of external NaCl. Five-day-old wild type and mutant seedlings were transferred onto the MS medium or MS supplemented with 5 mM, 25 mM and 50 mM NaCl or 50 mM NaNO_3_ or 50 mM KCl. Photographs were taken on the 14th day after the transfer; (**g**) length of primary roots and (**h**) fresh weight of seedlings on the 14th day after the transfer. Data are presented as the mean ± SE of triplicate experiments. Values labeled with different letters are significantly different (*p* < 0.05).

The original *sos3* mutant is an ethyl methane sulfonate (EMS)-mutagenized allele in which SOS3 (CBL4) lacks three amino acids [29]. Although this mutated version of CBL4 is impaired in Ca^2+^ binding, it is probably not a null allele, which may still have a residual function [30]. We thus isolated an independent T-DNA insertional *cbl4* allele in which the full-length transcript became undetectable by reverse transcription-polymerase chain reaction (RT-PCR) (Figure S1). As expected, *cbl4* is more sensitive than the original *sos3* mutant in 50 mM and 75 mM NaCl conditions (Figure 1). To investigate the functional interaction between CBL10 and three components of the SOS pathway (SOS1, CIPK24 and CBL4), a series of double mutants were created by genetic crossing and their genotypes were confirmed by RT-PCR (Figure S1). To test the salt sensitivity of the *cbl4 cbl10* mutant, five-day-old mutant and wild-type plants were transferred to the Murashige and Skoog (MS) medium with a series of NaCl concentrations. We found that *cbl4 cbl10* showed dramatically enhanced sensitivity to Na stresses than the single mutants in all tested concentrations of NaCl (5 mM, 25 mM and 50 mM), and the growth difference between the double and single mutant plants was much more pronounced as the Na^+^ level was elevated (Figure 2b–d). On the normal MS medium without NaCl, all mutants exhibited no differences from the wild type (Figure 2a). On the MS medium with 5 mM NaCl, the *cbl4 cbl10* double mutant already exhibited significant sensitivity with shorter root and smaller shoots as compared to the wild type, whereas the *cbl4* and *cbl10* single mutants did not show a significant difference from the wild-type plants (Figure 2b). When 25 mM NaCl was added to the medium, both *cbl4* and *cbl10* showed some sensitivity, but the *cbl4 cbl10* plants exhibited much more severe growth defects in both shoot and root tissues as compared to the *cbl4* or *cbl10* plants (Figure 2c). When the concentration of Na^+^ in the medium increased to 50 mM, the growth of the *cbl4* and *cbl10* plants was obviously inhibited compared to the wild-type plants, whereas the *cbl4 cbl10* plants could hardly survive after being treated for two weeks (Figure 2d). Quantitative analysis of the primary root length (Figure 2g) and fresh weight (Figure 2h) indicated that, compared with two single mutants, the *cbl4 cbl10* double mutant displayed more severe growth retardation as affected by the external Na^+^ in a dosage-dependent manner.

To determine whether the hypersensitivity of these mutants to NaCl is specifically attributable to Na^+^, we replaced 50 mM NaCl with 50 mM NaNO_3_ and 50 mM KCl and found that the mutant seedlings were sensitive to 50 mM NaNO_3_ but showed no difference in growth compared with the wild type on the medium containing 50 mM KCl (Figure 2e,f). This result indicates that the mutants were specifically sensitive to Na^+^ but not to K^+^ or Cl^−^. To extend the phenotypic analysis of the *cbl4 cbl10* mutants in mature plants, we also examined the salt sensitivity of the *cbl4 cbl10* mutant plants in hydroponic solutions with defined levels of external Na^+^ (Figure S2). We found that *cbl4 cbl10* grown in hydroponic conditions also showed dramatically enhanced sensitivity to Na^+^ stresses as compared to the single mutants. Under 15 mM Na^+^ conditions, the *cbl4 cbl10* plants were much more stunted than single mutants, as revealed also by the shoot biomass (Figure S2c,e). Taken together, these results demonstrated that CBL10 and CBL4 function additively in salt tolerance in Arabidopsis.

### 2.2. Genetic Interaction between CBL10 and CIPK24

**Figure 3:**
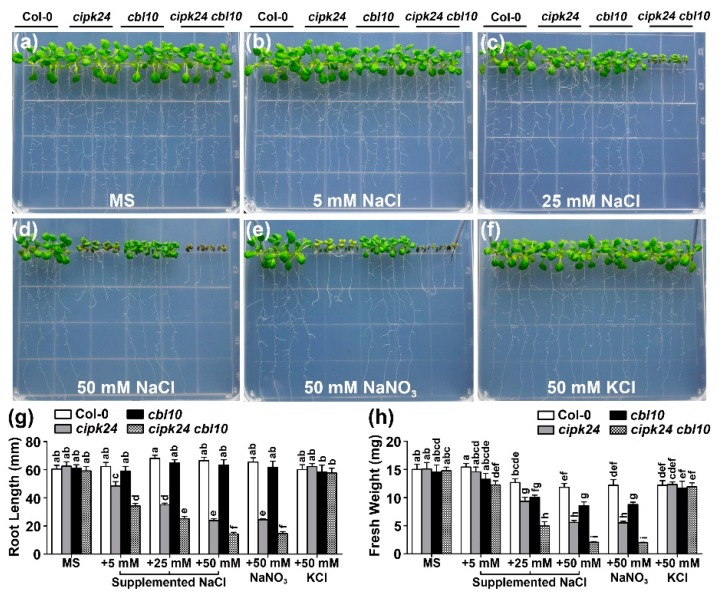
The *cipk24 cbl10* double mutant plants are more sensitive to external Na^+^ than the *cipk24* and *cbl10* single mutants. (**a–f**) Growth phenotype of Col-0, *cipk24*, *cbl10* and *cipk24 cbl10* under different concentrations of external salt. Five-day-old wild type and mutant seedlings were transferred onto the MS medium or MS supplemented with 5 mM, 25 mM and 50 mM NaCl or 50 mM NaNO_3_ or 50 mM KCl. Photgraphs were taken on the 14th day after the transfer; (**g**) length of primary roots and (**h**) fresh weight of seedlings on the 14th day after the transfer. Data are presented as the mean ± SE of triplicate experiments. Values labeled with different letters are significantly different (*p* < 0.05).

In earlier studies, both CBL4 and CBL10 have been shown to physically interact with CIPK24 [16,17]. However, it is not clear if the CBL10 functionally interacts with CIPK24. We examined the growth phenotype of the *cipk24 cbl10* double mutant under various external Na^+^ concentrations, in comparison with the wild type as well as the *cipk24, cbl10,* and *sos1* single mutants. On the normal MS medium without NaCl, all mutants exhibited no differences from the wild type (Figure 3a). After being grown in the MS agar medium supplemented with external NaCl for two weeks, both roots and shoots of the *cipk24 cbl10* seedlings were more severely stunted as compared with the *cipk24* or *cbl10* single mutant (Figure 3b–d). Measurement of the root length and shoot fresh weight verified the growth phenotypes (Figure 3g,h).

To extend the phenotypic analysis of the *cipk24 cbl10* double mutant in mature plants, we examined the salt sensitivity of the *cipk24 cbl10* mutant plants in hydroponic solutions with defined levels of external Na^+^ (Figure S3). In the absence of the NaCl treatment, none of the mutants showed any significant growth differences compared with the wild type (Figure S3a,f). After being grown in the hydroponic 1/6 MS medium supplemented with 15 mM NaCl for six days, *cipk24 cbl10* showed more salt-sensitive phenotype than *cipk24* or *cbl10*, as revealed by the shoot biomass (Figure S3c,e). The growth difference between *cipk24 cbl10* and the single mutant plants was much more pronounced after the plants were grown in the medium containing 5 mM NaCl for 25 days (Figure S3g). As illustrated in Figure S3g, the *cipk24 cbl10* leaves were chlorotic and could hardly bolt while the wild type and the single mutant plants successfully bolted and flowered.

**Figure 4:**
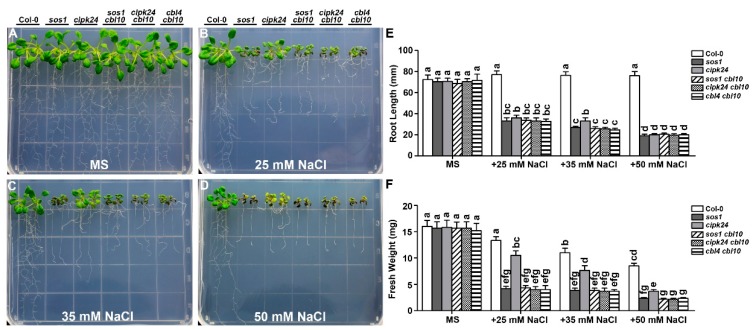
The *cipk24 cbl10* and *cbl4 cbl10* double mutants are more sensitive to external Na^+^ than the *cipk24* single mutant. (**a–d**) Five-day-old seedlings of each genotype were transferred onto the MS medium or MS supplemented with 25 mM, 35 mM and 50 mM NaCl. Photographs were taken on the 14th day after the transfer; (**e**) length of primary roots and (**f**) fresh weight of seedlings of each genotype on the 14th day after the transfer. Data are presented as the mean ± SE of triplicate experiments. Values labeled with different letters are significantly different (*p* < 0.05).

**Figure 5:**
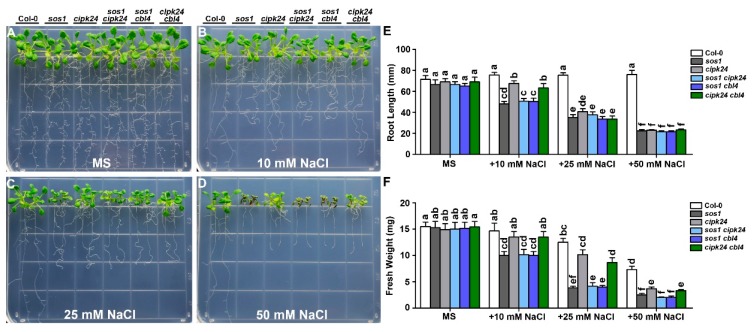
Phenotypic analysis of Na^+^ sensitivity in the *sos1 cipk24*, *sos1 cbl4* and *cipk24 cbl4* mutants. (**a–d**) Five-day-old seedlings of each genotype were transferred onto the MS medium or MS supplemented with 10 mM, 25 mM and 50 mM NaCl. Photographs were taken on the 14th day after the transfer; (**e**) length of primary roots and (**f**) fresh weight of seedlings of each genotype on the 14th day after the transfer. Data are presented as the mean ± SE of triplicate experiments. Values labeled with different letters are significantly different (*p* < 0.05).

On the standard MS medium without NaCl, the wild type and all the mutant plants grow normally and exhibited no differences (Figure 4a and Figure 5a). While the *cipk24 cbl10* double mutant showed more severe phenotype than each of the single mutant (Figure 3 and Figure 4b–f), the *cipk24 cbl4* double mutant showed similar phenotype to *cipk24* (Figure 5b–f). Therefore, the functional interaction between CBL10 and CIPK24, if any, was distinct from the relationship of CBL4 and CIPK24 that forms the linear pathway. In other words, CIPK24 probably functions as a major component downstream of the CBL4-mediated pathway, alternative or additional kinases other than CIPK24 are likely to be involved in the CBL10-mediated pathway. Among all the single and double mutants, *sos1* was the most severely affected with similar phenotype to all the double mutants combining *cbl10* and any SOS pathway mutant (Figure 4; Figure S4), suggesting that SOS1 may serve as a predominant determinant in plant salt tolerance or as a converging point for the CBL4 and CBL10-mediated pathways.

### 2.3. CBL10 and CBL4 Differentially Regulate Na^+^ and K^+^ Accumulation

**Figure 6:**
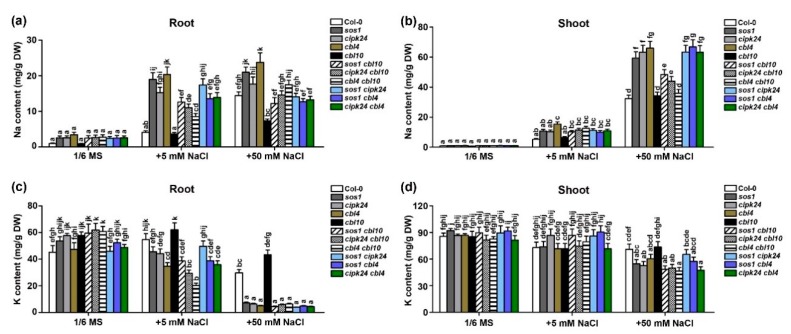
The Na^+^ and K^+^ content in the wild type (Col-0) and various mutants grown under different external Na^+^ conditions. Three-week-old wild type and mutant plants grown in hydroponic 1/6MS solutions containing 0, 5 mM or 50 mM NaCl for five days were harvested for the measurements of the Na^+^ and K^+^ contents. (**a**,**b**) The Na^+^ content in the root (**a**) and shoot (**b**) under different Na^+^ regimes; (**c**,**d**) the K^+^ content in the root (**c**) and in the shoot (**d**) under different Na^+^ regimes. Data are presented as the mean ± SE of triplicate experiments. Values labeled with different letters are significantly different (*p* < 0.05).

Maintenance of the K^+^/Na^+^ homeostasis within plant cells is critical for plant salt tolerance. The SOS pathway components are believed to maintain a low Na^+^ content and normal K^+^ content in the cytosol by exporting excessive Na^+^ back to the external space [4,31]. In contrast, CBL10 is hypothesized to sequester Na^+^ into the vacuole [16]. Since the whole-plant phenotype analysis did not provide a clear-cut answer regarding the relationship of the CBL10 and SOS pathway (Figure 4), we resorted to an analysis on the Na^+^/K^+^ homeostasis in the mutant plants to further delineate the relationship of the CBL10- and CBL4-directed pathways. Plants of various genotypes were grown ina normal hydroponic medium for three weeks and then transferred to the same medium or a medium containing 5 mM or 50 mM NaCl and cultured for five more days. The Na^+^ and K^+^ contents of roots and shoots were measured by inductively coupled plasma-mass spectrometry (ICP-MS). When grown in a medium without NaCl, no significant difference in the Na^+^ and K^+^ contents was found between the wild type and all the mutants in either roots or shoots. When grown in a medium containing 5 mM NaCl condition, the wild type and *cbl10* plants had similar Na^+^ and K^+^ contents in roots and shoots as those plants grown under control conditions without the NaCl supplement. In contrast, the SOS pathway mutants and all double mutants showed higher Na^+^ and lower K^+^ content in roots with no significant change in shoots. When grown under high salt conditions (50 mM NaCl), all plants of different genotypes displayed significantly increased Na^+^ content and decreased K^+^ content in both roots and shoots, as compared to those grown under control conditions. The *sos* mutants (*sos1*, *sos2*/*cipk24* and *sos3*/*cbl4*) accumulated much more Na^+^ and less K^+^ than the wild type in both roots and shoots (Figure 6), consistent with results in earlier studies [29,32]. In contrast to the *sos* mutants, *cbl10* contained less Na^+^ and more K^+^ in the roots than the wild type and approximately equal Na^+^ and K^+^ contents to the wild type in shoots, similar to results shown in a previous report [16]. To our surprise, in 50 mM NaCl condition, all three *cbl10*-associated double mutants, including *sos1 cbl10*, *cipk24 cbl10* and *cbl4 cbl10*, accumulated significantly less Na^+^ than the *sos* single mutants in the shoots albeit the Na^+^ content was still higher than that of the wild-type plants (Figure 6b). These results strongly suggested that CBL10 should regulate a transport process that is independent from the SOS1-mediated Na^+^ efflux. Otherwise, the salt content in the mutants should follow the same pattern when either the CBL10 pathway or SOS pathway is disrupted.

### 2.4. CBL10 and CBL4 Display Different Subcellular Localizations

**Figure 7:**
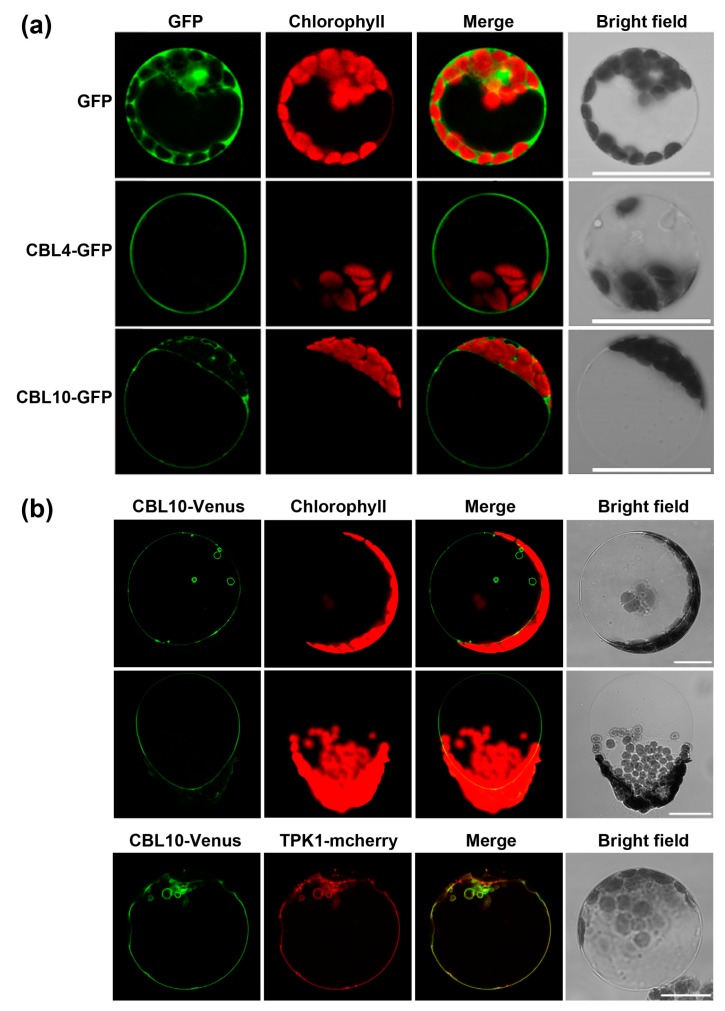
Subcellular localization of calcineurin B-like proteins 4 (CBL4) and CBL10. (**a**) Confocal laser scanning microscopy images of Arabidopsis mesophyll protoplasts transiently expressing either green fluorescent protein (GFP) alone or CBL4-GFP or CBL10-GFP fusion proteins under the control of the 35S promoter; (**b**) protoplasts generated from Agrobacterium-infiltrated *Nicotiana benthamiana* leaves expressing CBL10-Venus (upper row), a vacuole released from a mesophyll protoplast (middle row) and protoplasts co-expressing CBL10-Venus and TPK1-mcherry (lower row). The GFP/Venus signals (green), chloroplast auto-fluorescence/mcherry (red), merged signals from GFP/Venus and chlorophyll channels or from Venus and mcherry (yellow), and bright-field images are shown separately from left to right in each lane. Bars = 20 μm.

Tissue-specific expression pattern and subcellular locations of specific downstream partners including CIPKs underlies specificity for CBLs to mediate the stress response. We revisited the subcellular localization of CBL4 and CBL10 in different assay systems. In Arabidopsis mesophyll protoplasts, the empty green fluorescent protein (GFP) alone was ubiquitously distributed in the cell (Figure 7a). CBL4 is uniquely localized to the plasma membrane, while CBL10 is preferentially localized to the intracellular membranes including tonoplast (Figure 7a). In the epidermal cells of *N. benthamiana*, CBL10 is also predominantly localized to the vacuolar membrane (Figure 7b). Furthermore, when vacuoles were released from isolated mesophyll protoplasts of the transgenic plant, they showed a clear Venus, an enhanced yellow fluorescent protein, signal at the tonoplast (Figure 7b). On the other hand, the CBL10-Venus signal largely overlapped with the two-pore K^+^ channel 1 (TPK1)-mCherry fusion protein (Figure 7b), a tonoplast marker. In addition, we generated transgenic Arabidopsis lines that constitutively expressed the CBL10-Venus fusion protein in the *cbl10* mutant background. Importantly, the expression of CBL10-Venus complemented the salt-sensitive phenotype of *cbl10*, confirming the proper function of the fusion protein (Figure S6a–c). As observed in transiently transformed *N. benthamiana* cells, the Venus fluorescence in these plants was found to be intracellularly targeted (Figure S6d). Additionally, previous work in poplars suggested that the targeting of CBL10 to the tonoplast is required for salt stress adaptation [20]. Based on these results, we speculate that CBL10 serves as a calcium sensor protein probably in the vacuolar membrane. However, we cannot exclude the possibility that CBL10 may also be associated with other types of membrane in some specific cell types or under certain physiological conditions. Future studies should be directed to alternative approaches, such as the transmission electron microscopy, to conclusively determine the subcellular localization of CBL10 with higher resolution in plant cells.

## 3. Discussion

High concentrations of Na^+^ in the cytoplasm disrupt the ionic balance and the uptake of essential mineral nutrients, such as K^+^, which in turn causes adverse effects on many metabolic pathways [33,34]. To cope with salt stress, plants have evolved various tolerance mechanisms including two transport processes at the single cell level. Either exporting Na^+^ out of the cell, or compartmentalizing excessive Na^+^ into the vacuole [2]. These two transport mechanisms act in a coordinated manner to maintain a low Na^+^ concentration in the cytoplasm. However, it remains unknown if they are regulated by the same or different signaling pathways. The SOS pathway is generally viewed as a signaling mechanism for the activation of the Na^+^ efflux through SOS1, a NHX-type Na^+^/H^+^ exchanger in the plasma membrane [4,15,31]. The loss of function of SOS genes thus results in hypersensitivity to NaCl, coupled with the Na^+^ over-accumulation in the cytoplasm. On the other hand, some Na^+^/H^+^ exchangers (NHXs) are localized in the tonoplast and may be involved in transporting Na^+^ from the cytoplasm to the vacuole [35]. However, the exact role of different NHX isoforms responsible for salt tolerance remains unclear.

Interestingly, the two distinct but inter-connected salt transport processes appear to be both regulated by calcium signaling, in which calcineurin B-like proteins are thought to be the primary calcium sensors during salt stress adaptation. Among them, CBL4 and CBL10 display distinct tissue expression patterns and subcellular localizations [16,17]. The spatial specificity of these two calcium sensors may contribute to their functional diversification in salt stress adaptation. In order to understand how they work synergistically in the regulation of salt tolerance, we genetically analyzed the salt-sensitive phenotype of the *cbl4 cbl10* double mutant in comparison with the single mutants. The *cbl4 cbl10* double mutant was dramatically more sensitive to salt as compared to the *cbl10* and *cbl4* single mutants, suggesting that CBL4 and CBL10 either functionally overlap or each directs an independent salt-tolerance pathway. If the two CBLs are functionally overlapping, they should regulate the same transport processes and then the double mutant should not only show more severe phenotype but also show more severe deviation in the Na^+^ and K^+^ contents as compared to the wild-type plants. However, that was not the case: *cbl4* and *cbl10* displayed generally opposite Na^+^ and K^+^ profiles. Although the *cbl4 cbl10* double mutant plants showed Na^+^ over-accumulation compared to the wild type, but significantly lower Na^+^ content than the *cbl4* single mutant (Figure 6a,b). This suggests that CBL10 should not be involved in the CBL4-regulated Na^+^ extrusion process (activation of SOS1), although these two calcium sensors interact with a common downstream kinase CIPK24 (SOS2). Instead, CBL10 should regulate a distinct Na^+^-transport process in response to high salt, probably the Na^+^ sequestration into the vacuole, as suggested by its tonoplast localization and the lower Na^+^ content in the *cbl10* mutants. This is consistent with the general theme that the Na^+^ efflux or Na^+^ sequestration into the vacuole both contribute to salt tolerance and disrupting either may result in elevation of the Na level in the cytoplasm and thus leading to salt sensitivity. Certainly disrupting both transport processes would lead to more severe salt sensitivity, which match the more sensitive phenotype of *cbl4 cbl10*.

Previous studies suggested that CIPK24 serves as the common downstream target of CBL4 and CBL10 by forming CBL4-CIPK24 or CBL10-CIPK24 complex at the plasma or vacuolar membrane separately [16,17,18]. Although our findings in this study supported this hypothesis, they also suggested that other CIPKs, in addition to CIPK24, should be also involved in the CBL10-mediated pathway based on the genetic evidence that double mutants of *cbl4 cbl10* and *cipk24 cbl10* displayed a significant enhancement in Na^+^ sensitivity as compared to *cipk24* (Figure 4). Indeed, screened by the yeast two-hybrid assay, we found that CBL10 did interact with other CIPKs in addition to CIPK24 (Figure S5). Various combinations of CBL10 with different CIPKs may target different target proteins and exhibit diverse functions.

To examine whether SOS1 is a downstream component of CBL10 in the pathway, we also compared the salt sensitivity between *sos1 cbl10* and *sos1*. In our test conditions, the salt sensitivity of *cbl4 cbl10* and *sos1 cbl10* was comparable to *sos1* (Figure 4 and Figure S4), suggesting that SOS1 may serve as a converging point for the two CBL pathways. However, the double mutants *cbl4 cbl10* and *sos1 cbl10* accumulated much lower Na^+^ content than the single mutants of *cbl4* and *sos1,* respectively, under salt conditions (Figure 4), which implies that CBL10 and SOS1 functions in two different transport processes in regulating Na^+^ homeostasis. For instance, in the *sos* single mutants in which the Na^+^ efflux is blocked, the CBL10 pathway functions to transport Na^+^ into the vacuole leading to the over-accumulation of Na^+^ in plant tissues. When the vacuole sequestration is defective in the *cbl10*-associated double mutants, the Na^+^ uptake is inhibited as a feedback of lacking storage space, leading to less accumulation and thus lower Na^+^ content in these double mutants as compared to the *sos* single mutants (Figure 6). Despite overall lower Na^+^ content in plant tissues, the double mutants showed similar salt sensitivity as *sos1* because the majority of Na^+^ in these double mutants is in the cytoplasm effectively causing toxicity. Our results thus provide an example where a two-tier evaluation system must be implemented for dissecting salt tolerance mechanism in plants: First by whole-plant phenotyping (general tolerance) and further by the analysis of Na^+^/K^+^ homeostasis (detailed transport processes).

Concerning the target transporters for CBL10, all evidence so far supports the hypothesis that the CBL10-CIPK pathway may regulate Na-transporters in the tonoplast [16,18] (Figure 6 and Figure S2). Sequestration of Na^+^ into the vacuole is presumably fulfilled by an array of Na ^+^ transporters that include the vacuole-localized NHX-type Na^+^ (K^+^)/H^+^ transporters. However, recent genetic evidence indicates that vacuole-localized antiporters NHX1-4 have Na^+^-transport activities but may not contribute much to the vacuolar Na^+^ compartmentation, because the quadruple knockout mutant *nhx1/2/3/4* is not more sensitive to NaCl than the wild type [36]. Furthermore, vacuoles isolated from the quadruple mutant still retain the Na^+^ uptake that is independent to the pH gradient, implicating the presence of NHX-independent Na^+^ transporters in *Arabidopsis* vacuoles [36]. We speculate that some of these unknown transporters may serve as CBL10-CIPK targets. On the other hand, endosomal compartments emerge as critical players that may be directly involved in controlling Na^+^ homeostasis [37,38,39]. A possible but yet to be proved model is that the Na^+^ sequestration into the plant vacuole may actually be achieved, at least in part, through endosomal Na^+^ scavenging processes and subsequent fusion to the vacuole. NHX5 and NHX6 are localized to endosomal compartments and associated with protein trafficking from the Golgi/Trans-Golgi Network (TGN) to vacuoles. Supporting this hypothesis is the finding that disruption of two endosomal NHXs in the *nhx5 nhx6* double mutant showed increased sensitivity to salinity [38]. Considering the fact that a proportion of the CBL10 protein was also localized to the dynamic endosomal compartments [16], NHX5/6 could also act as the candidate targets of the CBL10-CIPK complexes. In a recent work, translocon of the outer membrane of the chloroplasts 34 (TOC34) was identified as a novel interaction partner protein of CBL10 at the outer membrane of chloroplasts, clearly indicating that CBL10 can relay Ca^2+^ signals in more diverse ways than currently known [40]. Identification of target transporter(s) directly regulated by the CBL10-CIPK module is an important and challenging task for future research, which would also unravel the pathway through which Na^+^ is deposited into the plant vacuole.

## 4. Materials and Methods

### 4.1. Plant Materials

*Arabidopsis thaliana* ecotype Columbia (Col-0) were used as the wild type in this study. The T-DNA insertion mutants *sos1* (SALK_060960), *cipk24* (SALK_016683), *cbl4* (SALK_113101) and *cbl10* (SALK_056042) were obtained from the Arabidopsis Biological Resource Center (Columbus, OH, USA). Mutants with multiple gene-knockout events were generated by genetic crosses, and homozygous mutant plants were screened from F2 generation and identified by genomic PCR using primers listed in Supplementary Table S1. The *sos2* and *sos3* mutants were in the *gl1*/*gl1* Col-0 background and the mutation *gl1* did not affect the salt tolerance phenotype [17].

### 4.2. Growth Conditions and Stress Rreatment

For culture on agar plates, seeds of different genotypes were sterilized with 75% ethanol for 10 min, washed in sterilized water three times, and sown on the Murashige and Skoog (MS) medium containing 2% sucrose (Sigma, St. Louis, MO, USA) and 0.8% phytoblend (Caisson Labs, Smithfield, UT, USA). The plates were incubated at 4 °C in darkness for two days and then were positioned vertically in the growth chamber at 22 °C under 12 h light/12 h dark photoperiod. After germination, five-day-old seedlings were transferred onto agar-solidified MS media supplemented with Na^+^ at the indicated concentrations (5, 10, 25, 35, 50, 75 or 100 mM NaCl) and were grown at 22 °C under 12 h light/12 h dark photoperiod. For hydroponic culture, after germination and being grown on the MS plate for ten days, the seedlings were transferred to 1/6-strength MS liquid solutions and were grown under the 12 h light/12 h dark photoperiod in the plant growth chamber. Fresh liquid solutions were replaced once a week. After two-week culture, the plants were treated with 1/6 MS solutions supplemented with a range of NaCl concentrations (5, 15 or 25 mM NaCl) and were grown under 12 h light/12 h dark photoperiod.

### 4.3. Measurements of Na^+^ and K^+^ Content

Wild type and mutant plants were fed with the 1/6-strength MS solution for three weeks and were transferred to the 1/6 MS solutions containing 0, 5 or 50 mM NaCl. After the five-day treatment, seedlings of each genotype were collected and pooled into shoots and roots. The samples were washed with ultrapure water four times, dried at 80 °C for 48 h, milled to fine powder, weighed, and digested with concentrated HNO_3_ (Sigma-Aldrich, Milwaukee, WI, USA) in 100 °C water bath for 1 h. Na^+^ and K^+^ concentrations were determined using an ICP mass spectrometer (PerkinElmer NexION 300, Waltham, MA, USA). The operating parameters were as follows: Nebulizer Gas Flow STD/KED: 1.02 L/min, Auxiliary Gas Flow: 1.02 L/min, Plasma Gas Flow: 17 L/min, ICP RF Power: 1300 W, Analog Stage Voltage: −1750 V, Pulse Stage Voltage: 950 V, Discriminator Threshold: 12V, Defector Voltage: −12 V, Quadrupole Rod Offset STD: 0 V, Cell Entrance Voltage STD/KED: −6 V/−4 V, Cell Exit Voltage STD/KED: −6 V/−26 V, Cell Rod Offset STD/KED: −16 V, Axial Field Voltage KED/DRC: 475 V/200 V. The inductively coupled plasma-mass spectrometry (ICP-MS) was calibrated by direct analysis of external calibration standards containing known concentrations of the analytes. Each sample was tested three times.

### 4.4. Subcellular Localization Studies

Two Arabidopsis mesophyll protoplasts were prepared from four-week-old rosette leaves by soaking leaf slices with an enzymatic mixture containing 1% Cellulase R10 and 0.4% Macerozyme R10 (Yakult Pharmaceutical, Tokyo, Japan) for 2–3 h. The digested protoplasts were re-suspended in the W5 medium (154 mM NaCl, 125 mM CaCl2, 5 mM KCl and 2 mM MES, pH 5.8) and transfected in combination with 20 μg recombinant plasmids (CBL4-GFP and CBL10-GFP) by the PEG-mediated transformation protocol [41]. The transformed protoplasts were incubated in dark at 22 °C overnight before imaging using laser scanning confocal microscope (Leica TCS-SL, Buffalo Grove, IL, USA). Infiltration of *Nicotiana benthamiana* leaves was performed as previously described by Walter et al. (2004) [41]. Protoplasts were prepared three days after infiltration by cutting leaf discs into small pieces and incubating for 3 h in the enzyme solution (0.4 M mannitol, 1% cellulase R10, 0.3% macerozyme R10, pH 5.7). The filter settings are Ex 488 nm/Em 475–560 nm for GFP, Ex 514 nm/Em 490–560 nm for Venus, Ex 561 nm/Em 540–640 nm for mcherry, and Ex 488 nm/Em 650–720 nm for chlorophyll.

### 4.5. Statistical Analysis of the Data

All data in this work were obtained from three independent experiments and 20 individual plants of each genotype in each treatment conditions were used in each replicate. Data were subjected to statistical analyses using the one-way analysis of variance (ANOVA) followed by Duncan’s multiple range test (*p* < 0.05).

## Supplementary Materials

Supplementary materials can be found at https://www.mdpi.com/1422-0067/20/10/2421/s1. Figure S1: Molecular identification of various double mutants. Figure S2: Phenotypic analysis of Na^+^ sensitivity in *cbl4 cbl10* under hydroponic conditions. Figure S3: Phenotypic analysis of Na^+^ sensitivity in *cipk24 cbl10* under hydroponic conditions. Figure S4: Phenotypic analysis of Na^+^ sensitivity in the *sos1 cbl10* double mutant plants under mild salt conditions. Figure S5: CBL10 and CBL4 shared several interacted CIPKs in common. Figure S6: CBL10-Venus fusion proteins were primarily targeted to intracellular membranes and can complement the salt sensitivity of the *cbl10* mutant. Table S1: Primers used in this study.
