# Supplementary material for: Calcineurin B-Like Proteins CBL4 and CBL10 Mediate Two Independent Salt Tolerance Pathways in Arabidopsis
Source: Int J Mol Sci. 2019 May 16;20(10):2421. doi: 10.3390/ijms20102421 (PMC6566158; doi:10.3390/ijms20102421)
Supplement: Supplementary file 1 [file ijms-20-02421-s001.zip › CBL10 Supplementary Materials.docx]

**Supplementary Materials**

Supplementary Methods

*Yeast two-hybrid assay*

The yeast two-hybrid assay was performed using GAL4 two-hybrid system. To construct bait plasmids, the vector pGBKT7 fused with the coding region of CBL4 or CBL10 was used. The prey plasmids were constructed from the vector pGADT7, which encodes the coding region of CIPKs. Direct interaction of two proteins was investigated by co-transformation of the respective plasmid constructs into yeast strain AH109 according to manufacturer’s instructions (Clontech). The positive clones were arrayed with serial dilutions on SD/-Leu/-Trp/-His selection medium containing 2mM 3AT for the isolation of false positive interactions, and further confirmed on SD/-Leu/-Trp/-His/-Ade selection medium containing X-α-gal for testing the LacZ gene activity.


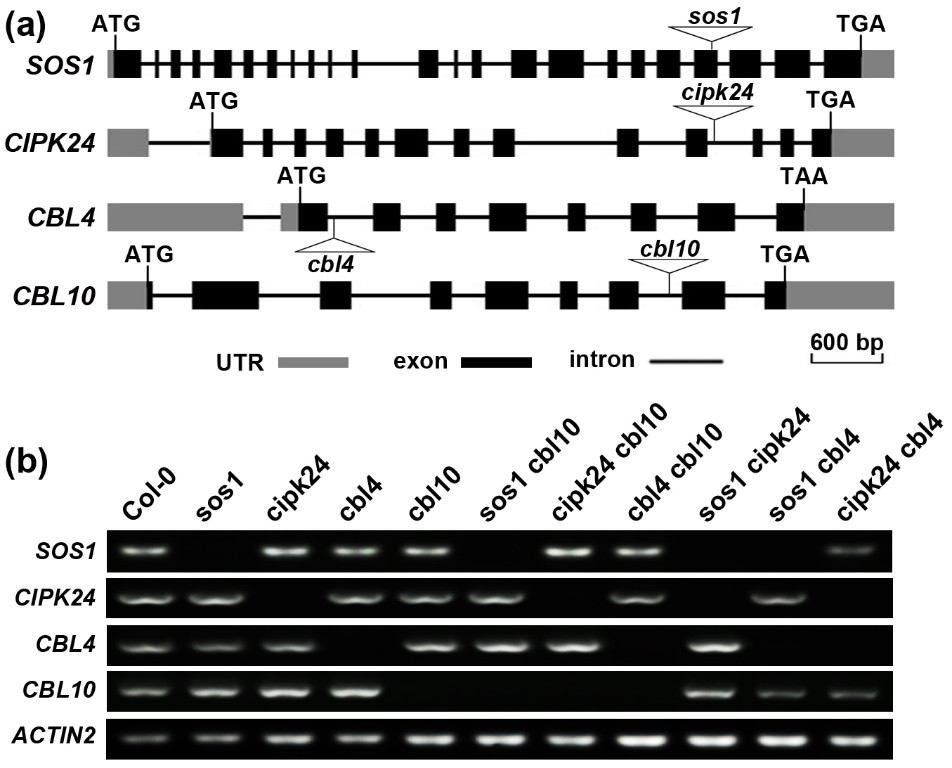


**Figure S1.** Molecular identification of various double mutants. (a) Schematic diagram of the T-DNA location in *sos1*, *cipk24*, *cbl4* and *cbl10* mutants. Black boxes and lines respectively represent exons and introns to scale, and the 5’ and 3’ UTRs are shown as shaded boxes. The position of the T-DNA insertion is indicated by the triangle.

(b) RT-PCR analysis of *SOS1*, *CIPK24*, *CBL4* and *CBL10* transcripts in the wild type (Col-0) and various mutants. Expression of *ACTIN2* is shown as an internal control.

**
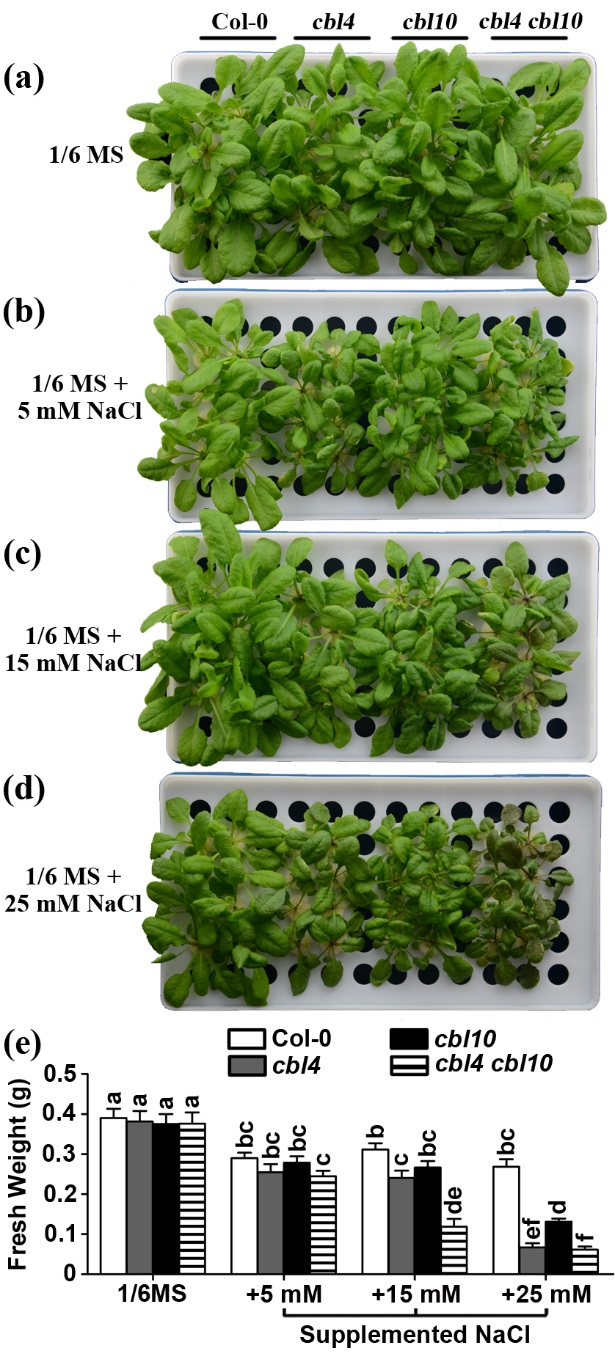
**

**Figure S2.** Phenotypic analysis of Na^+^ sensitivity in *cbl4 cbl10* under hydroponic conditions. Two-week-old seedlings of each genotype were transferred onto hydroponic 1/6 MS medium or 1/6 MS supplemented with 5 mM, 15 mM and 25 mM NaCl. Photographs were taken on the 6th day (a-d) after the transfer. (e) Fresh weight of seedlings of each genotype on the 6th day after the transfer. Data are presented as the mean ± SE of triplicate experiments. Values labelled with different letters are significantly different (*P* < 0.05).


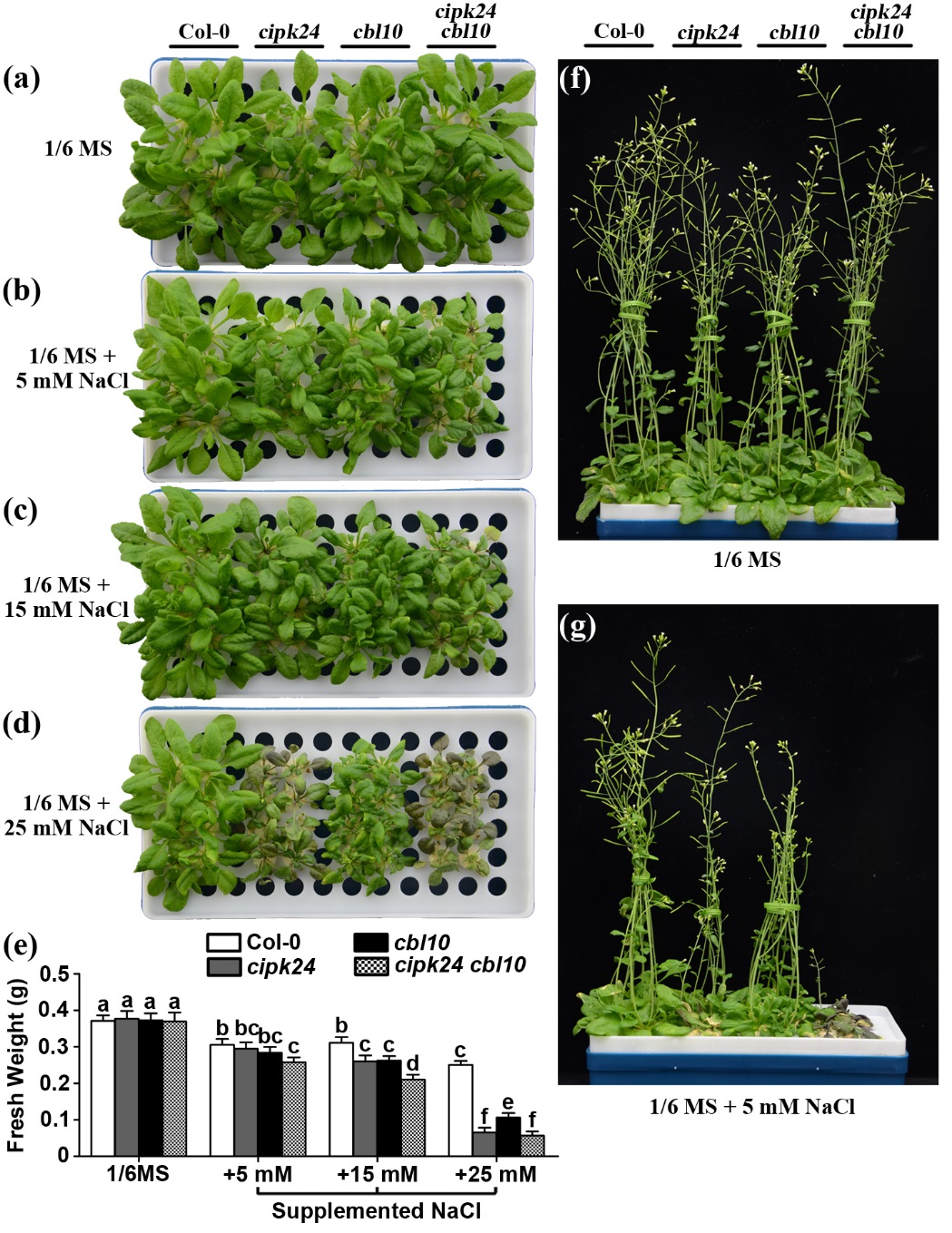


**Figure S3.** Phenotypic analysis of Na^+^ sensitivity in *cipk24 cbl10* under hydroponic conditions. Two-week-old seedlings of each genotype were transferred onto hydroponic 1/6 MS medium or 1/6 MS supplemented with 5 mM, 15 mM and 25 mM NaCl. Photographs were taken on the 6th day (a-d) or 25th day (f-g) after the transfer. (e) Fresh weight of seedlings of each genotype on the 6th day after the transfer. Data are presented as the mean ± SE of triplicate experiments. Values labelled with different letters are significantly different (*P* < 0.05).


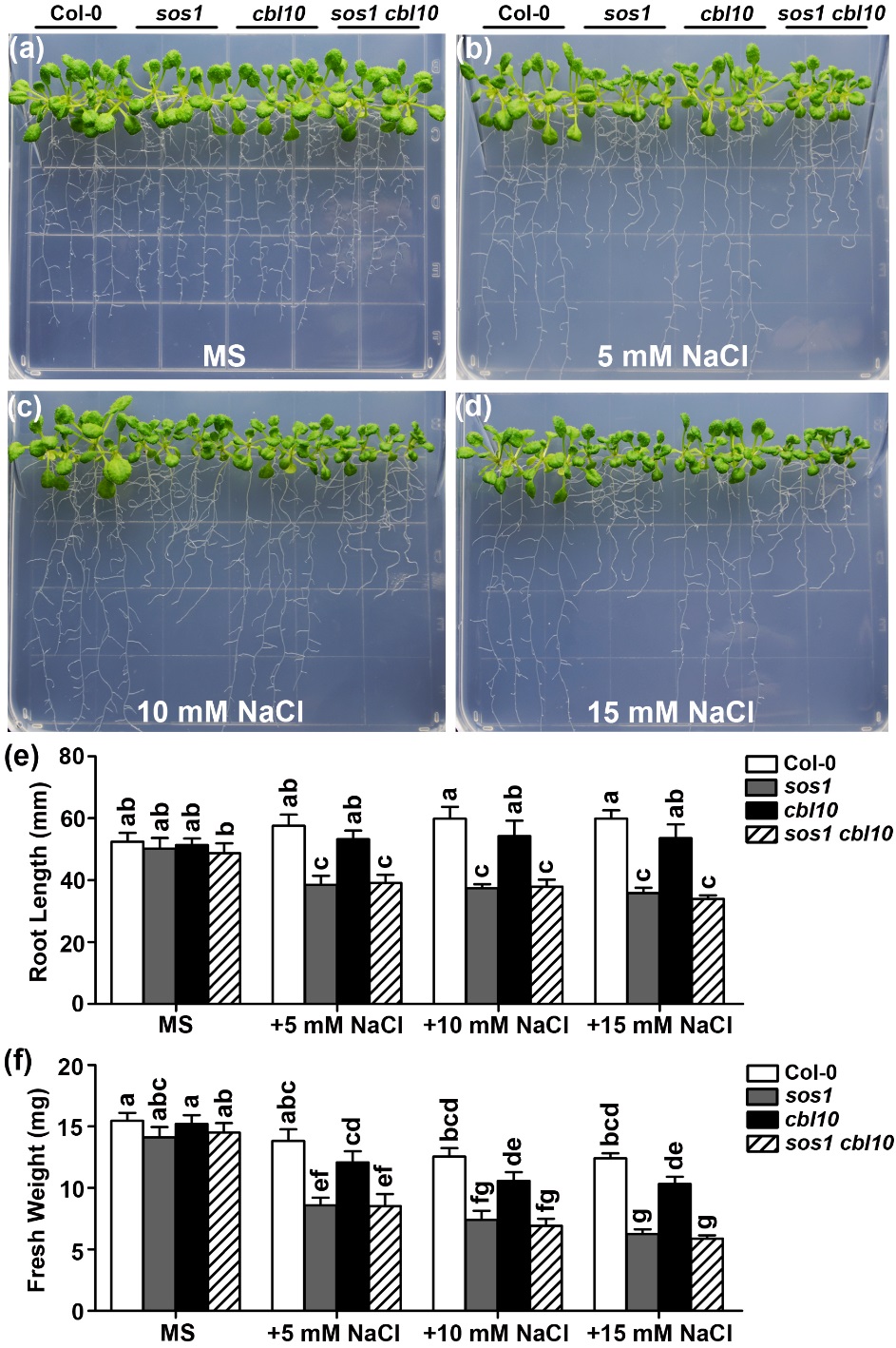


**Figure S4.** Phenotypic analysis of Na^+^ sensitivity in the *sos1 cbl10* double mutant plants under mild salt conditions. (a-d) Growth phenotype of Col-0 and *sos1 cbl10* under different concentrations of external NaCl. Five-day-old Col-0 and *sos1 cbl10* seedlings were transferred onto MS medium or MS supplemented with 5 mM, 10 mM and 15 mM NaCl. Photographs were taken on the 14th day after the transfer. (e) Length of primary roots and (f) fresh weight of wild-type and mutant plants on the 14th day after the transfer. Data are presented as the mean ± SE of triplicate experiments. Values labelled with different letters are significantly different (*P* < 0.05).


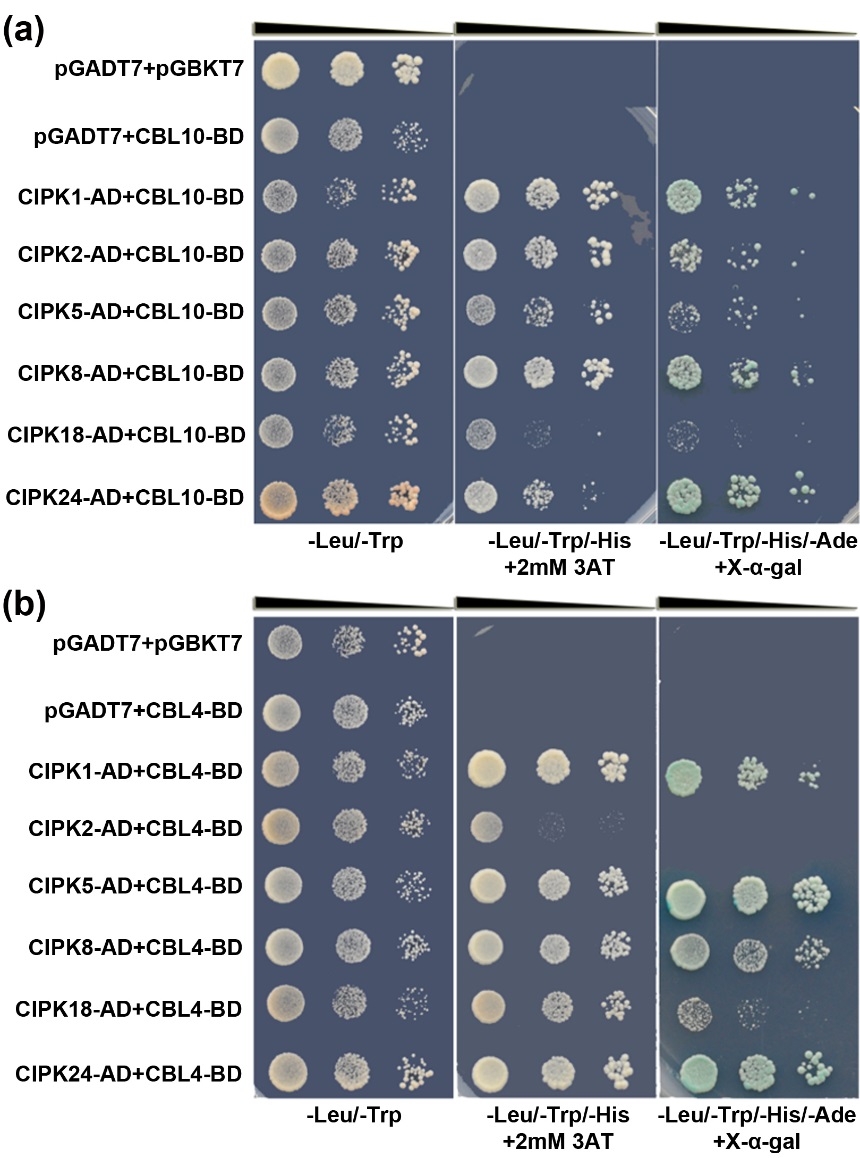


**Figure S5.** CBL10 and CBL4 shared several interacted CIPKs in common. Yeast two-hybrid assay of the interactions between CBL10 (a) or CBL4 (b) and different CIPKs. Yeast AH109 cells were co-transformed with various combinations of BD- and AD-fusion constructs as indicated. Serial decimal dilutions of corresponding yeast cells were spotted onto selective SD medium without Leu and Trp as a control (left panel) or onto the selective SD medium lacking Leu, Trp, and His for monitoring growth (middle panel) or onto the selective medium lacking Leu, Trp, His and Ade for a more stringent assay (right panel). Photographs were taken after cultivation at 30 °C for 3 days.


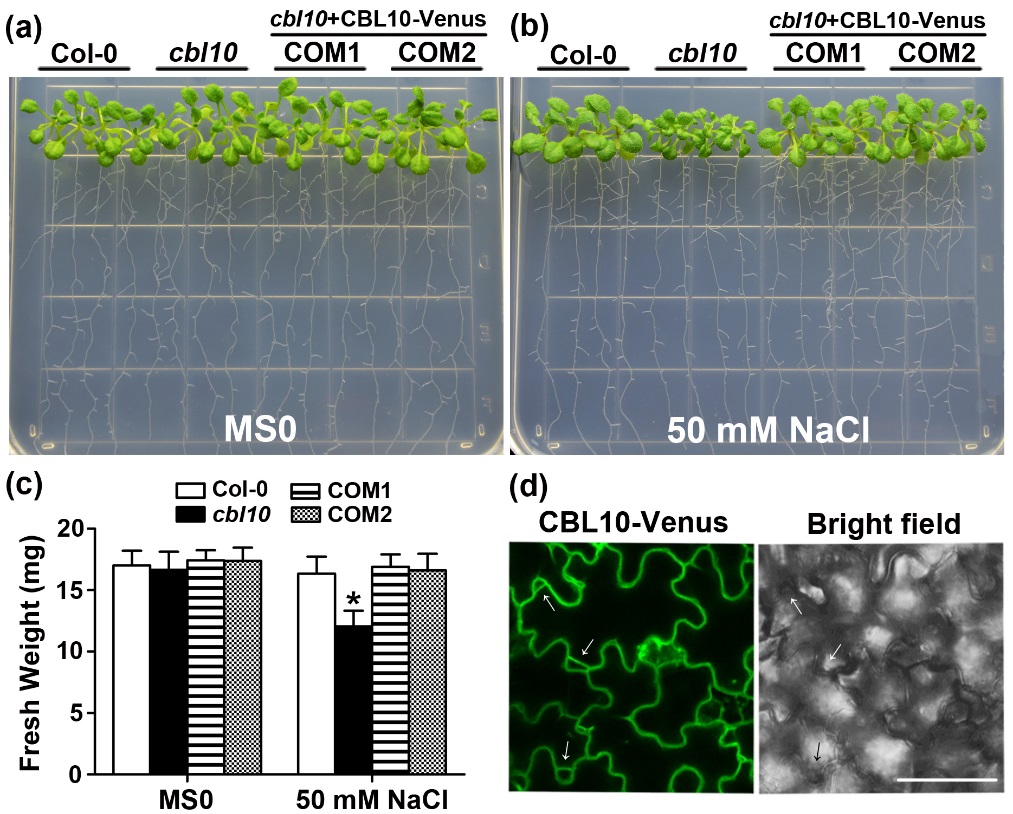


**Figure S6.** CBL10-Venus fusion proteins were primarily targeted to intracellular membranes and can complement the salt sensitivity of the *cbl10* mutant. (a-b) Five-day-old Col-0, *cbl10* and two lines of *cbl10*+CBL10-Venus (COM1 and COM2) were transferred to MS medium or MS supplemented with 50 mM NaCl. Photographs were taken on the 14th day after the transfer. (c) Fresh weight measurements in (a) were performed two weeks after transfer. Graphs depict mean ± SE of triplicate experiments. (d) Subcellular localization of CBL10 in *cbl10*+CBL10-Venus seedlings. The photographs depict leaf epidermis cells of the Arabidopsis cotyledons. Arrows indicate a membraneous invagination forming the nuclear pocket. Bars=50 μm.

**Table S1.** Primers used in this study.

| Purpose | Name | Primer sequence (5’ to 3’) |
| --- | --- | --- |
| *sos1* mutant  identification and RT-PCR | SOS1-RTF | AATCACTCGCTGCATCCAACTT |
|  | SOS1-RTR | CGATTTCGATTGATTCTCCCGTC |
| *cipk24* mutant  identification and RT-PCR | CIPK24-RTF | CCCCTGATGATGAATGCCTTTG |
|  | CIPK24-RTR | TCACCAGCAGCCTTTCTTACGTC |
| *cbl4* mutant  identification and RT-PCR | CBL4-RTF | ATGGGCTGCTCTGTATCGAAG |
|  | CBL4-RTR | CACCTAAGGACCGGACAAATTC |
| *cbl10* mutant  identification and RT-PCR | CBL10-RTF | TGCATCAATCCAGGAGAAGACC |
|  | CBL10-RTR | CTTCTTTCCTCGTTCTGCTCTTG |
| *ACTIN2*  RT-PCR | ACTIN2-RTF | GGAAGGATCTGTACGGTAAC |
|  | ACTIN2-RTR | GGACCTGCCTCATCATACT |
| T-DNA left  border primer | LBa1 | TGGTTCACGTAGTGGGCCATCG |
