# Supplementary figures and images for: Calcineurin B-Like Proteins CBL4 and CBL10 Mediate Two Independent Salt Tolerance Pathways in Arabidopsis
Source: Int J Mol Sci. 2019 May 16;20(10):2421. doi: 10.3390/ijms20102421 (PMC6566158; doi:10.3390/ijms20102421)

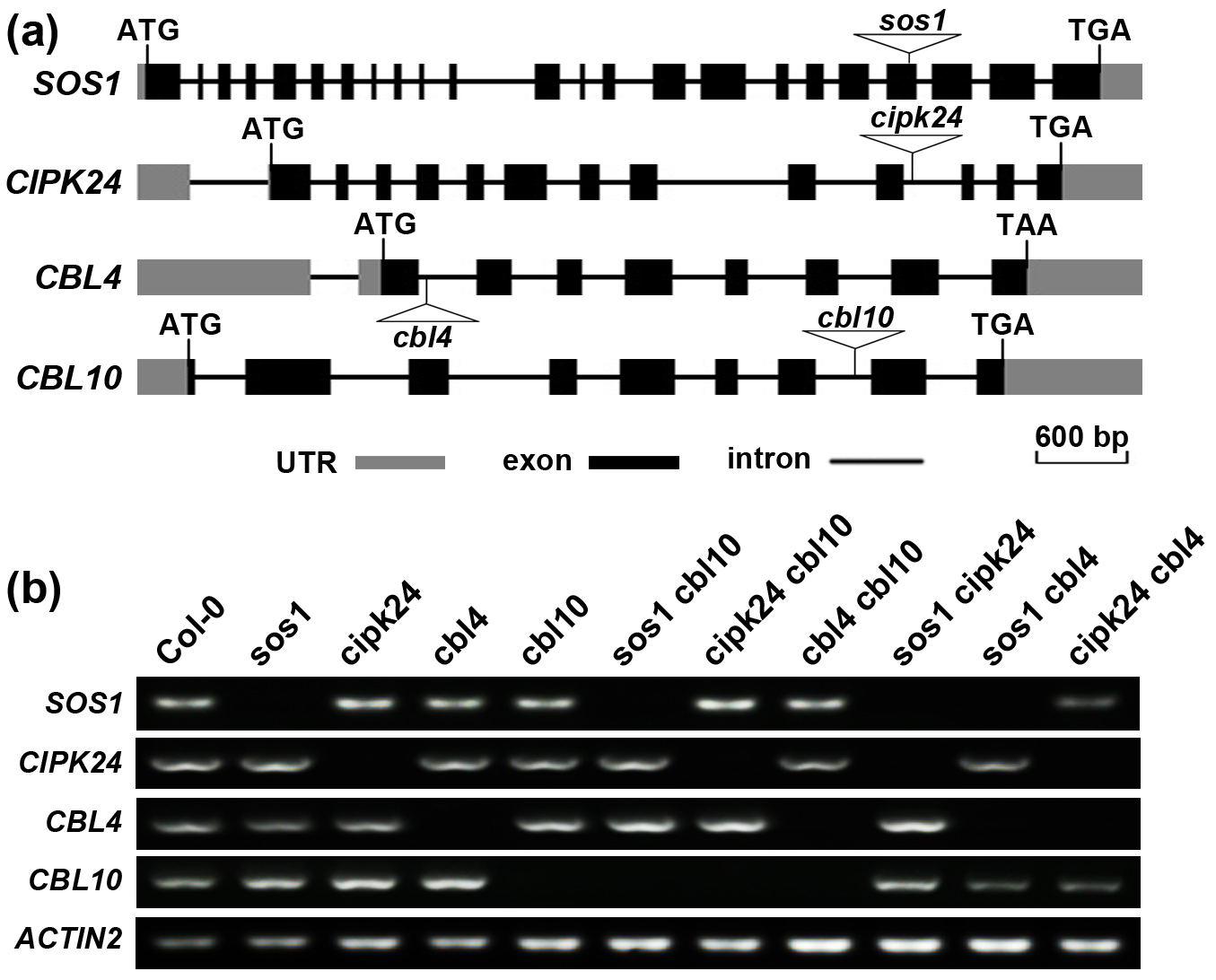

Supplement: Supplementary file 1 [file ijms-20-02421-s001.zip › Figure S1.jpg]

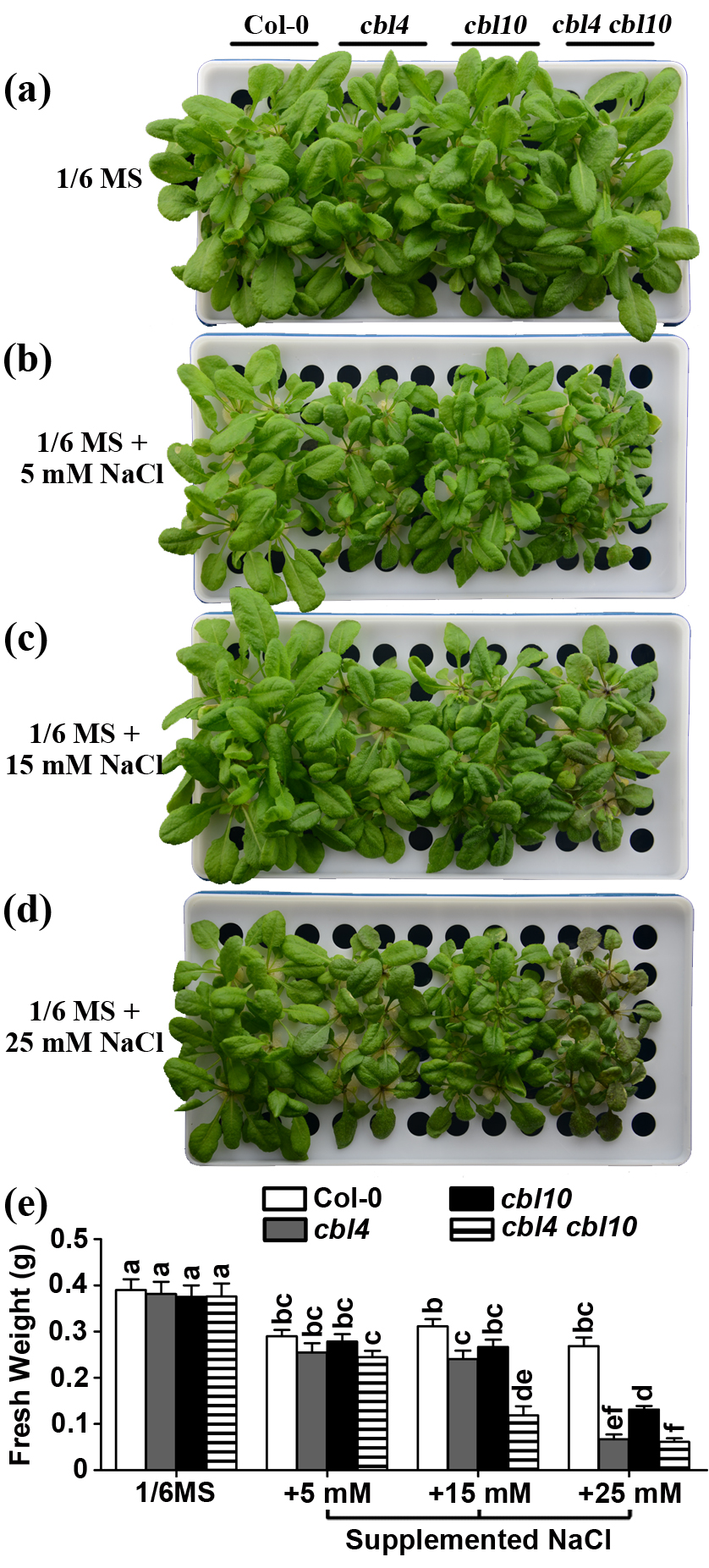

Supplement: Supplementary file 1 [file ijms-20-02421-s001.zip › Figure S2.jpg]

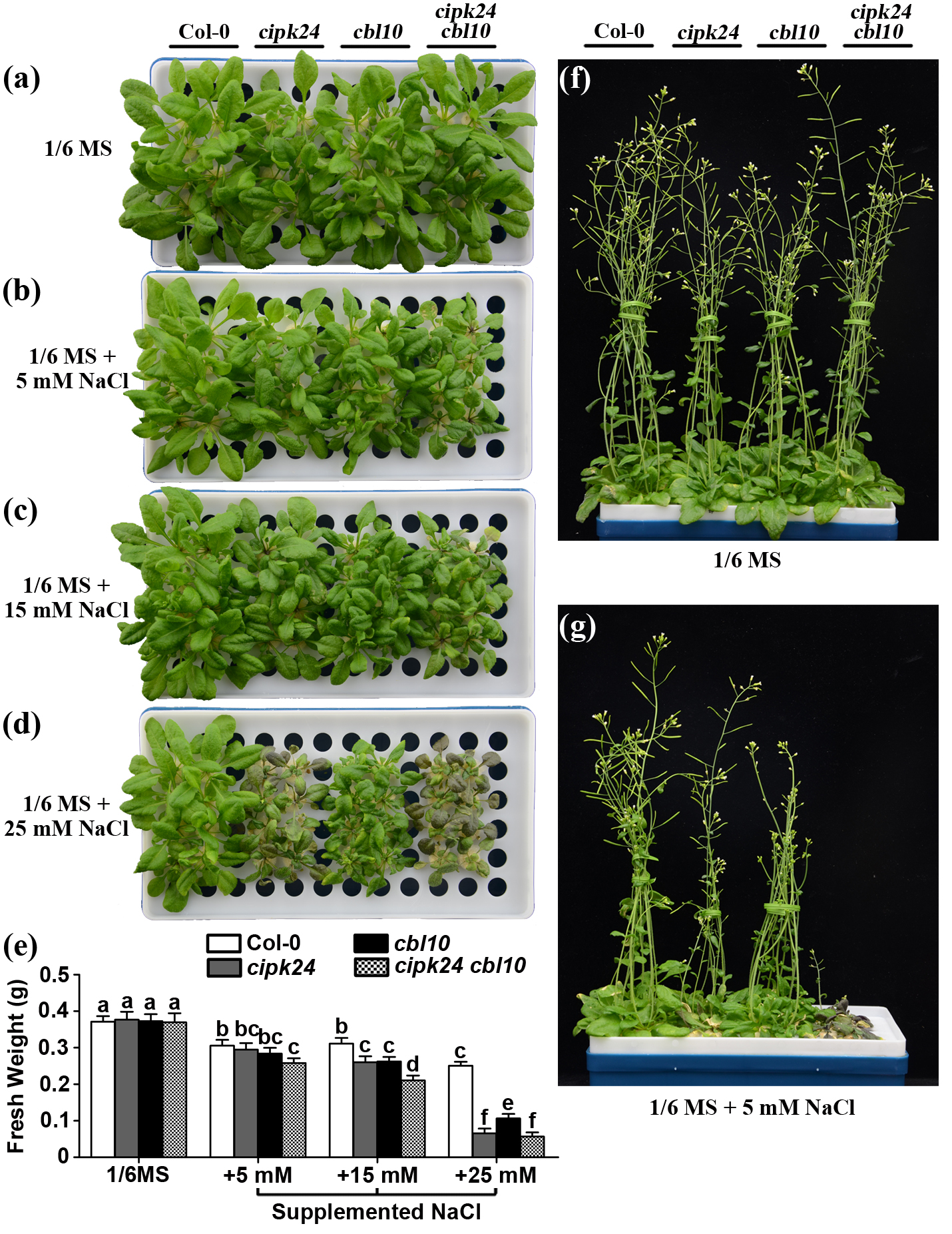

Supplement: Supplementary file 1 [file ijms-20-02421-s001.zip › Figure S3.jpg]

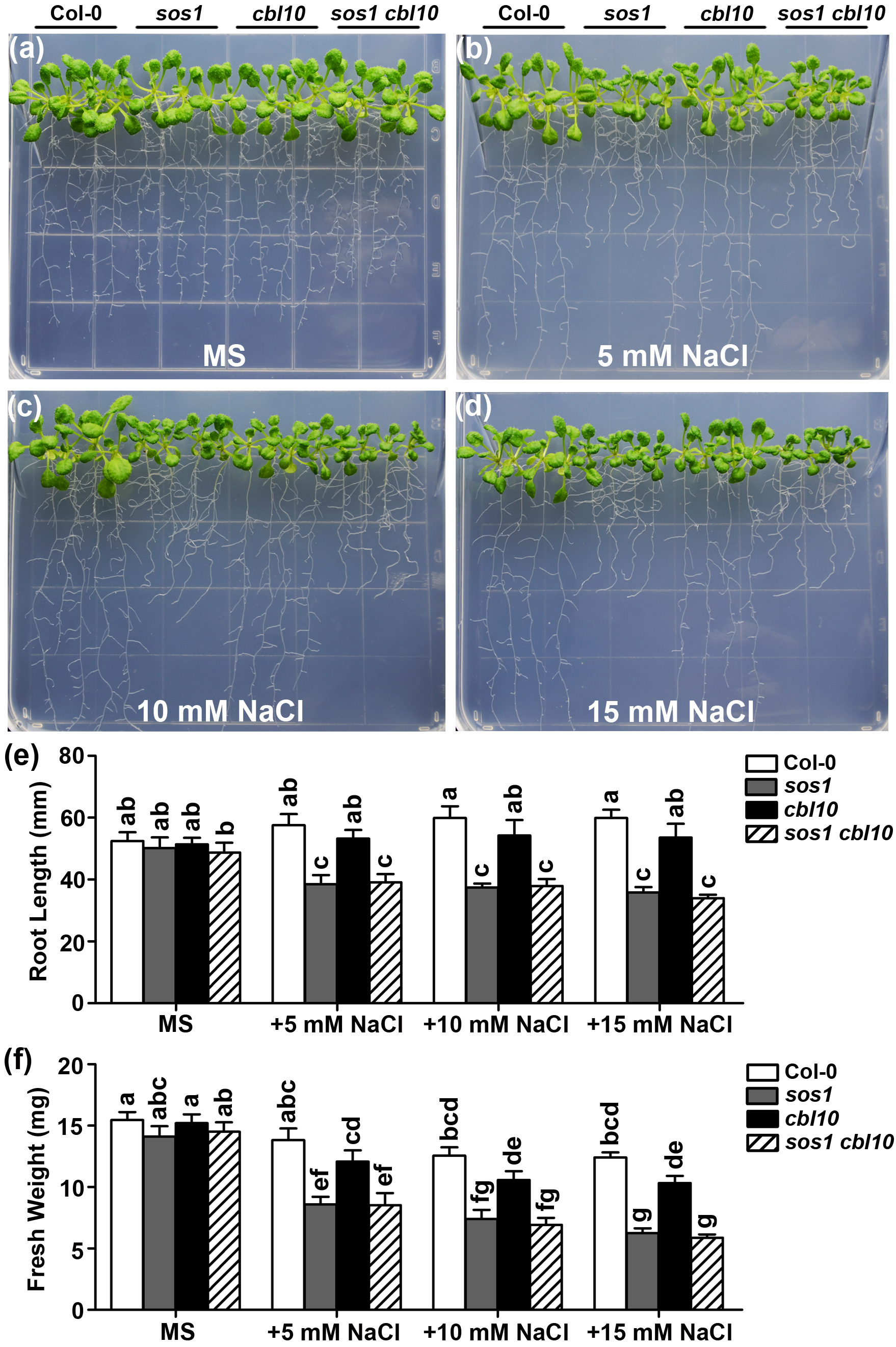

Supplement: Supplementary file 1 [file ijms-20-02421-s001.zip › Figure S4.jpg]

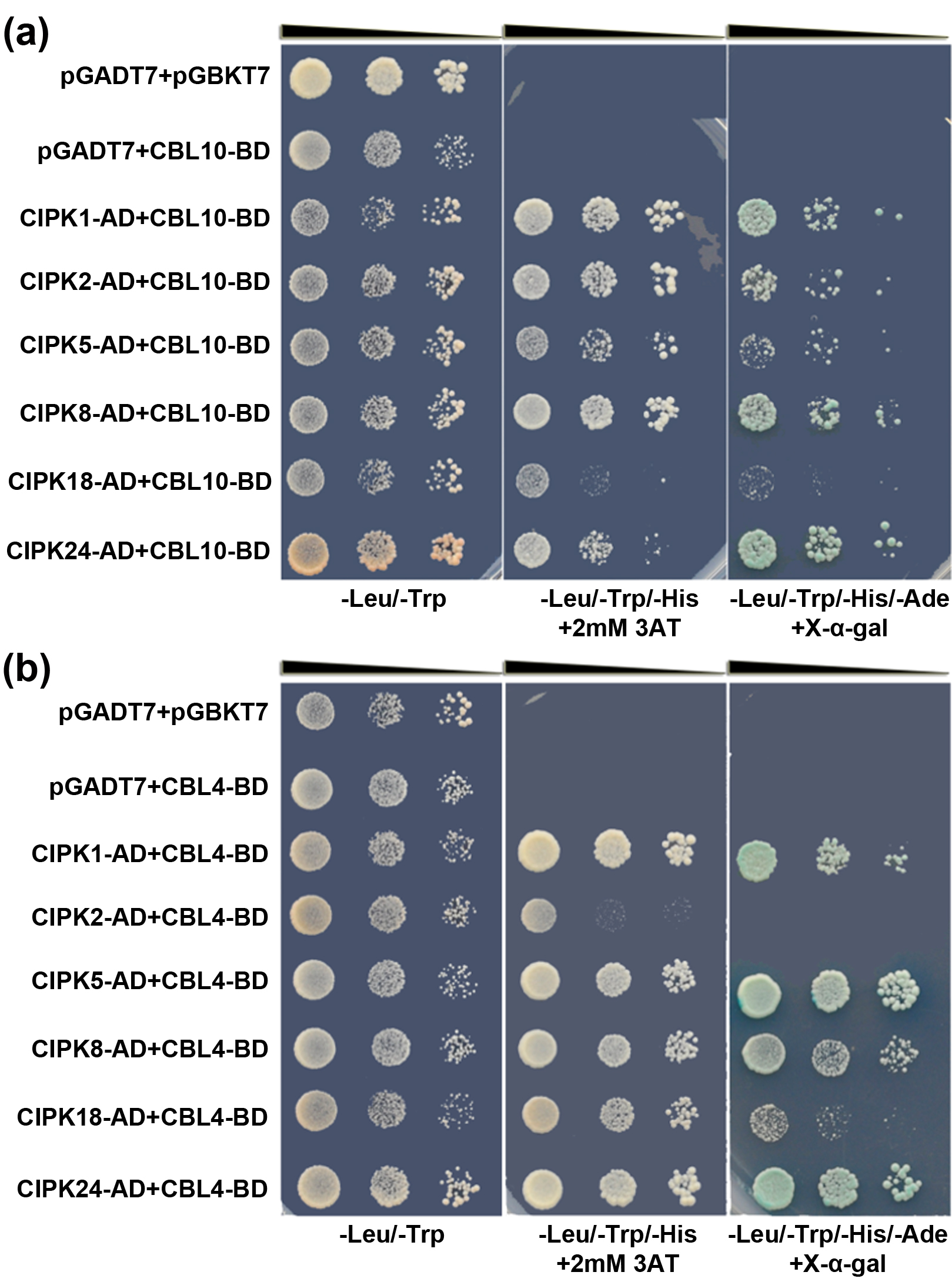

Supplement: Supplementary file 1 [file ijms-20-02421-s001.zip › Figure S5.jpg]

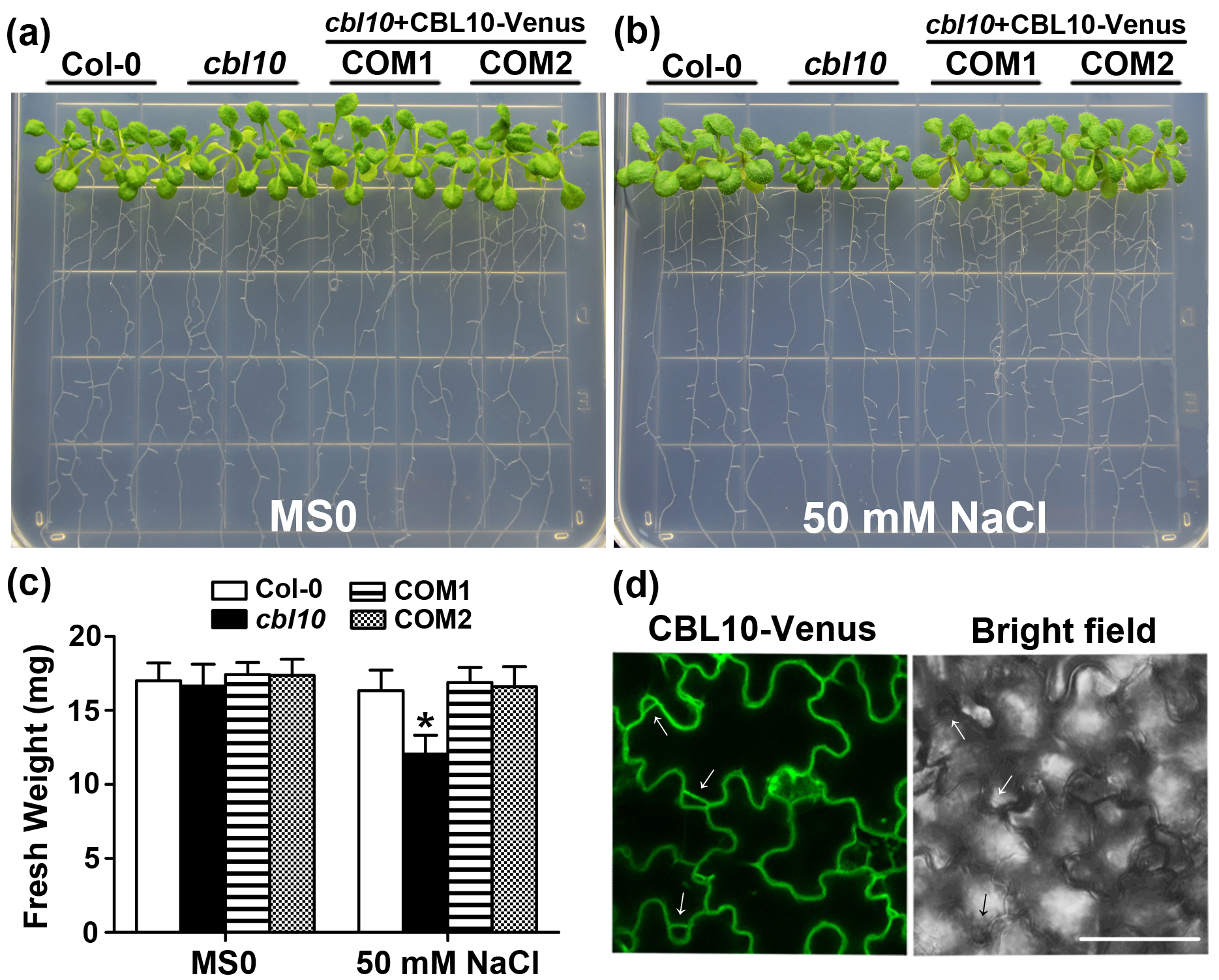

Supplement: Supplementary file 1 [file ijms-20-02421-s001.zip › Figure S6.jpg]
